# Case Report: Cerebrovascular Events Associated With Bacterial and SARS-CoV-2 Infections in an Adolescent

**DOI:** 10.3389/fneur.2021.606617

**Published:** 2021-04-08

**Authors:** Charles de Marcellus, Laurent Dupic, Charles-Joris Roux, Imane El Aouane El Ghomari, Perrine Parize, Romain Luscan, Florence Moulin, Manoelle Kossorotoff

**Affiliations:** ^1^Pediatric Intensive Care Unit, APHP University Hospital Necker-Enfants Malades, Paris, France; ^2^Université de Paris, Paris, France; ^3^Pediatric Radiology Department, APHP University Hospital Necker-Enfants Malades, Paris, France; ^4^Pediatric Department, CH André Mignot Versailles, Le Chesnay, France; ^5^Department of Infectious Diseases and Tropical Medicine, APHP University Hospital Necker-Enfants Malades, Paris, France; ^6^Pediatric Otorhinolaryngology Department, APHP University Hospital Necker-Enfants Malades, Paris, France; ^7^French Center for Pediatric Stroke, Pediatric Neurology Department, APHP University Hospital Necker-Enfants Malades, Paris, France; ^8^Inserm U1266, Paris, France

**Keywords:** Covid-19, SARS-CoV-2, stroke, cerebral venous thrombosis, Lemierre syndrome, cerebral vasculitis

## Abstract

Neurologic manifestations associated with Covid-19 are increasingly reported, especially stroke and acute cerebrovascular events. Beyond cardiovascular risk factors associated with age, some young adults without medical or cardiovascular history had stroke as a presenting feature of Covid-19. Suggested stroke mechanisms in this setting are inflammatory storm, subsequent hypercoagulability, and vasculitis. To date, a handful of pediatric stroke cases associated with Covid-19 have been reported, either with a cardioembolic mechanism or a focal cerebral arteriopathy. We report the case of an adolescent who presented with febrile meningism and stupor. Clinical, biological, and radiological features favored the diagnosis of Lemierre syndrome (LS), with *Fusobacterium necrophorum* infection (sphenoid sinusitis and meningitis) and intracranial vasculitis. The patient had concurrent SARS-CoV-2 infection. Despite medical and surgical antimicrobial treatment, stroke prevention, and venous thrombosis prevention, he presented with severe cerebrovascular complications. Venous thrombosis and stroke were observed, with an extension of intracranial vasculitis, and lead to death. As both *F. necrophorum* and SARS-CoV-2 enhance inflammation, coagulation, and activate endothelial cells, we discuss how this coinfection may have potentiated and aggravated the usual course of LS. The potentiation by SARS-CoV-2 of vascular and thrombotic effects of a bacterial infection may represent an underreported cerebrovascular injury mechanism in Covid-19 patients. These findings emphasize the variety of mechanisms underlying stroke in this disease. Moreover, in the setting of SARS-CoV-2 pandemic, we discuss in what extent sanitary measures, namely, lockdown and fear to attend medical facilities, may have delayed diagnosis and influenced outcomes. This case also emphasizes the role of clinical assessment and the limits of telemedicine for acute neurological condition diagnosis.

## Introduction

Neurologic complications of Covid-19 are increasingly reported, especially stroke and acute cerebrovascular events. In large series, SARS-CoV-2 infection appears to be associated with an increased risk of ischemic stroke, up to 4.5%, and this complication may be associated with an increased mortality risk (1, 2). Beyond cardiovascular risk factors associated with age, some young adults without medical or cardiovascular history had stroke as a presenting feature of Covid-19 (3). Suggested stroke mechanisms in this setting are notably inflammatory storm, subsequent hypercoagulability, and vasculitis (4). Children are less frequently infected by SARS-CoV-2 and usually have less severe forms of Covid-19 than adults (5–7). Regarding pediatric stroke occurrence, a handful of pediatric stroke cases associated with Covid-19 have been reported so far, with stroke attributed to embolic or hemodynamic mechanism (8, 9), focal cerebral arteriopathy (FCA) (10, 11), or bilateral vasculitis (12).

We report the case of an adolescent with bacterial meningitis, Lemierre syndrome (LS), and concurrent SARS-CoV-2 infection, with fatal cerebrovascular complications. This case suggests other stroke mechanisms that are scarcely described and presented in the pediatric population, such as a potential direct aggravating role of SARS-Cov-2 in the setting of a severe bacterial infection, and an indirect aggravating role of sanitary measures in the occurrence and severity of these cerebrovascular events.

## Case Description

**Figure 1:**
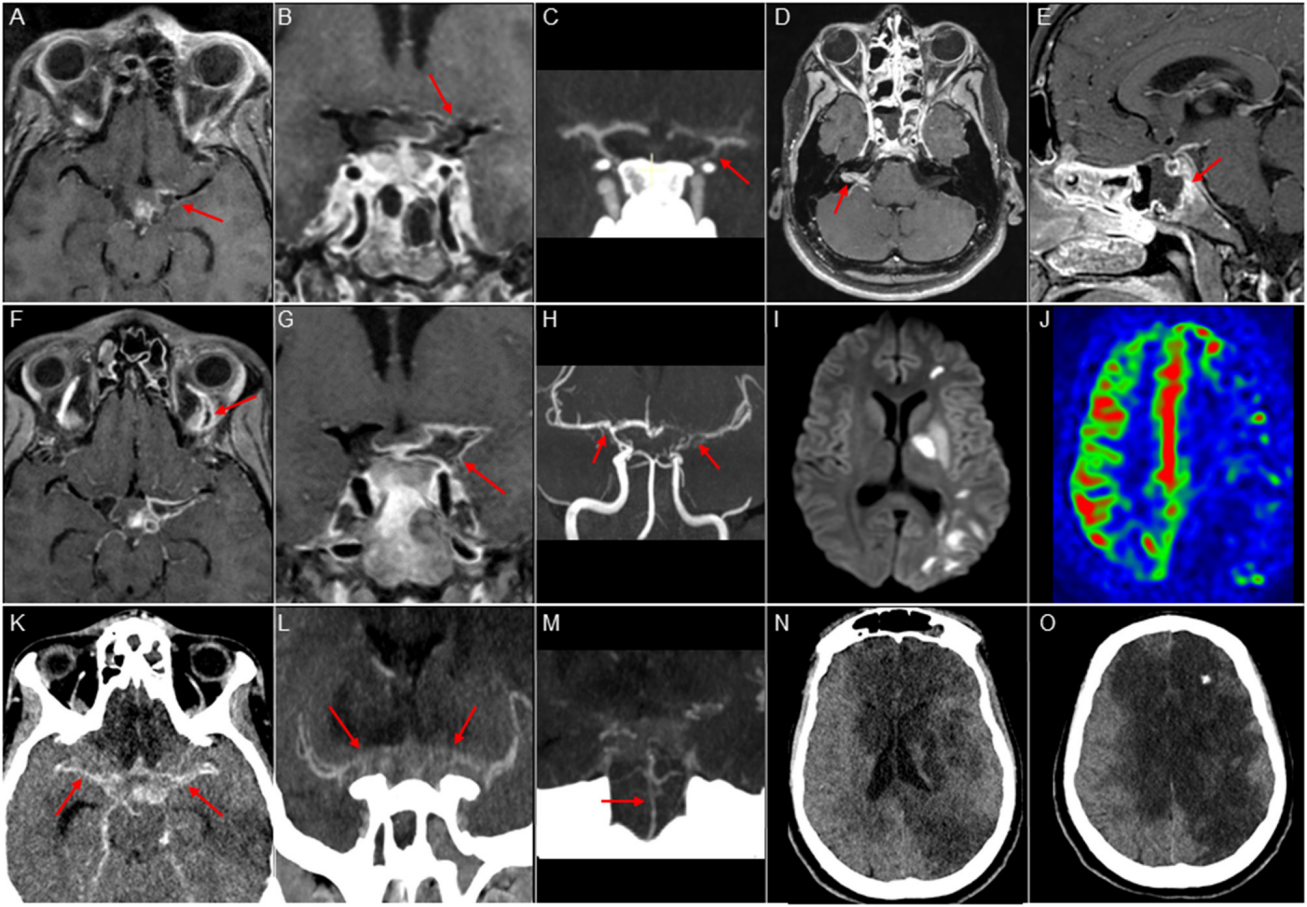
Imaging features. Upper panel: MRI and CT scan at admission. **(A,B,D,E)**: Cube T1 FatSat post Gadolinium contrast, axial **(A,D)**, coronal **(B)**, and sagittal **(E)** views. **(C)**: Contrast-enhanced CT with arterial phase, coronal view. Vasculitis of the Circle of Willis: stenosis of the left internal carotid artery, middle cerebral artery, and anterior cerebral artery **(A-C)**, with vessel wall enhancement **(A,B)**. Right acoustic-facial neuritis (**D**, arrow), sphenoid sinusitis with adjacent osteitis (**E**, arrow). Medium panel: MRI after sudden right hemiplegia and aphasia. **(F,G)** Cube T1 FatSat post Gadolinium contrast, axial view; **(H)** 3D Time-of-Flight angio-MR coronal view; **(I)** DWI sequence, axial view; **(J)** Arterial Spin Labeling perfusion sequence, axial view. Recent arterial ischemic stroke in the left middle cerebral territory **(I)** with extended hypoperfusion **(J)**, worsening of severe and bilateral vasculitis **(F-H)**, and left ophthalmic vein thrombosis (**F**, arrow). Lower panel: CT scan at day 14. **(K)**: post-contrast axial view; **(L,M)** angio-CT coronal view; **(N,O)**: axial view. Worsening of the intracranial vasculitis **(K-M)**, with basilar artery involvement **(M)**. Large bilateral infarcts in anterior and middle cerebral artery territories **(N,O)**.

A 16-year-old male of Caribbean descent without medical history presented with febrile neck stiffness, stupor, brisk reflexes, and no respiratory symptom, after 8 days of fever. Serum inflammatory studies showed elevated white blood cell count (12.6 G/L), elevated C-reactive protein (250 mg/L), procalcitonin (86 μg/L), and interleukin-6 (15.6 pg/mL, normal <8.5 pg/mL) levels. Hematology data showed initial thrombopenia (76 G/L), elevated fibrinogen (4.8 g/l), and D-dimer (1,357 ng/mL) levels. CSF analysis after receiving one cefotaxime injection favored bacterial meningitis with purulent fluid (1,566 cells/μl, 76% neutrophils), low glucose level (27 mg/dL), and elevated protein level (500 mg/dL). CSF Gram staining revealed both Gram-positive cocci and Gram-negative bacilli, without possible further identification; culture was sterile. CSF meningitis/encephalitis PCR panel and SARS-CoV-2 PCR were negative. Nasopharyngeal PCR was positive for SARS-CoV-2. Blood culture was positive for *F. necrophorum* and *Streptococcus constellatus*. Brain MRI revealed vasculitis of the Circle of Willis, with vessel wall enhancement and stenosis of the left internal carotid artery, middle cerebral artery (MCA), and anterior cerebral artery, associated with sphenoid sinusitis (Figure 1). The association of a sphenoid sinusitis with anaerobic germ and an intracranial arteritis, but without initial cervical or cerebral vein involvement, oriented toward a diagnosis of atypical LS, prompting antimicrobial treatment, stroke prevention, and venous thrombosis prevention. Antimicrobial management associated an intravenous combined antibiotic drug course adapted to microbiological findings and meningeal and bone diffusion (cefotaxime, metronidazole, and levofloxacin) with endoscopic sphenoidotomy achieving good drainage. Stroke prevention combined anti-inflammatory (dexamethasone pulse course) and antithrombotic (aspirin) treatments, associated with careful maintenance of hemodynamic homeostasis. The patient had daily echocardiography and transcranial Doppler assessments to adjust blood volume and blood pressure, in order to maintain adequate cerebral perfusion. Venous thrombosis prevention relied on prophylactic dose enoxaparin treatment. While still in the pediatric intensive care unit, the patient presented with sudden right hemiplegia and aphasia 9 days after admission. MRI confirmed recent arterial ischemic stroke in the left MCA territory, severe and bilateral vasculitis, and left ophthalmic vein thrombosis (Figure 1). Despite anti-inflammatory and antithrombotic treatment intensification with high-dose methylprednisolone pulse course, tocilizumab (anti-IL-6 receptor), remdesivir, and full-dose enoxaparin, clinical situation deteriorated, and the patient became comatose with severe brainstem dysfunction. While intubated, the patient had systematic standard chest X-Rays and arterial blood gases; all were normal. Intracranial pressure monitoring revealed intractable intracranial hypertension and the patient died 17 days after admission.

## Discussion

This adolescent presented a dramatic LS associating progressive intracranial vasculitis, subsequent arterial stroke, and venous thrombosis, leading to death despite maximal treatment. LS complicates an acute cervical or oropharyngeal bacterial infection. Its extended definition comprises head/neck venous thrombosis but also cervical and/or intracranial arterial vasculitis, potentially leading to stroke, especially in younger children and adolescents (13, 14). Septic embolism is also reported. In immunocompetent children, *F. necrophorum* is classically associated with head and neck infection-related LS. LS is a potentially severe condition. In a recent literature analysis at individual patient level including 712 patients with LS, global mortality reached 4% for the 2000-2017 period. It decreased to 2.3% for the 2012-2016 period, probably because of earlier diagnosis and better management, usually associating medical (antibiotic, anti-inflammatory, and antithrombotic treatments) and surgical therapies (14, 15). Among reported patients with LS, cerebrovascular complications (stroke, brain ischemia, brain edema, or herniation) occurred in 18/712 patients (2.5%). The majority (12/18) were children, adolescents, or young adults. Stroke was most often associated with systemic spread of the infection, especially pulmonary injury (11/14). Mortality related to cerebrovascular complications concerned only 0.8% (6/712) of patients with LS but 33% of patients with cerebrovascular complications. Death occurred early in the disease course (median 3 days after LS diagnosis) (14). Our patient, an immunocompetent adolescent, had a similar devastating disease course despite maximal treatment. Nevertheless, he presented atypical features, with slower disease progression and prominent vasculitis, which has been scarcely reported. We suggest that the coinfection with SARS-CoV-2 may have played a direct and indirect role in this evolution.

First, concurrent bacterial and viral infections may have synergistic effects. This process is well-described when pathogens may target the same organ(s). In the setting of Covid-19, coinfection with a viral or bacterial (*Mycoplasma pneumoniae)* respiratory pathogen is frequently found in children (16), and may represent a risk factor of severe disease or poor outcome (17, 18). Our patient had no respiratory involvement and synergistic effects through mutual potentiation of common pathophysiological processes may also be hypothesized. Beyond *F. necrophorum*'s reported affinity to endothelial cells and effects on coagulation (19, 20), well-described SARS-Cov-2 direct pro-inflammatory and pro-coagulant effects may represent additional risk factors for cerebrovascular events in the setting of LS. In patients with Covid-19, marked inflammatory status has been associated with stroke (3), and vasculitis related to endothelial cell infection *via* ACE2 receptors has been observed, with endothelial damage and increased subintimal inflammation, followed by hemorrhage or thrombosis (4, 21). Diffuse endothelial inflammation leading to ischemia was demonstrated in several organs including kidney, small bowel, and lung (22). Hyperinflammation can also lead to hypercoagulability, immunothrombosis through the upregulation of neutrophil extracellular traps, and formation of immune complexes. Patients with Covid-19 frequently display hypercoagulability (23) and an increased rate of thrombus, especially pulmonary embolism, encouraging antithrombotic prophylactic treatment (24). Cerebral venous thrombosis or stroke with large artery thrombus were also reported (3). In line with these findings, we hypothesize a synergistic effect of *F. necrophorum* and SARS-CoV-2 infections in stroke and venous thrombosis occurrence, and in the severity of the intracranial vasculitis observed in our case.

This coinfection potentiation suggests a different stroke mechanism from those hypothesized in the previously reported pediatric stroke cases associated with SARS-CoV-2. Two children had a FCA presumably associated with SARS-CoV-2 (10, 11), including one with vessel wall enhancement. FCA is a pediatric entity frequently observed during or after viral infection, in which the infection is supposed to act as a trigger of a localized self-limited inflammatory course, usually without notable systemic inflammation. Therefore, one should not be surprised to observe FCA with SARS-CoV-2, as it is observed with varicella, herpesviruses, viral respiratory pathogens, etc. (25). Two children had stroke complicating pediatric multisystem inflammatory syndrome temporally associated with COVID-19 (PIMS-TS) or multisystem inflammatory syndrome in children (MIS-C), a pediatric condition related to uncontrolled inflammatory response and cytokine storm during or following infection with SARS-CoV-2 (8). One had refractory shock and extracorporeal membrane oxygenation (ECMO) support; stroke occurence in the anterior and middle cerebral arteries was probably related to thromboembolic disorders related to ECMO (8). One recent report of a 9-year-old patient with PIMS-TS with aseptic meningitis, stroke, and bilateral internal carotid arteries stenosis suggested SARS-CoV-2 associated vasculitis (12). Our case goes beyond, rather suggesting a runaway of inflammation and hypercoagulation, leading to diffuse and progressive vasculitis, multiple strokes, but also cerebral venous thrombosis. Interestingly, another case may suggest the same mechanism, reporting a 2 year 7-month-old girl with tuberculosis meningitis and Covid-19, complicated by arterial ischemic stroke and cerebral venous thrombosis (26). Tuberculosis meningitis is known to predispose to arterial vasculitis and stroke, but this particularly severe evolution seems similar to our case. These findings emphasize the severity of SARS-CoV-2 coinfection, with a possible mechanism of “super-infection” (27), especially in the setting of a bacterial infection predisposing to cerebrovascular events. Of note, discrimination between causal relationship and incidental comorbidity remains difficult and it is currently unclear whether the infection *per se* represents an independent stroke risk factor (28).

Second, an indirect aggravating role of SARS-Cov-2 might be discussed, related to diagnostic delay. Appropriate therapy, but also timely diagnosis, is crucial in the management of LS (14). In our patient, the ongoing epidemic situation might have played an aggravating role. Retrospectively, at symptom onset the patient was in quarantine at home with his family, as his mother had proven Covid-19 infection. The first probabilistic diagnosis issued by his general practitioner after phone assessment because of headaches and fever was Covid-19; maintaining quarantine was recommended. This might have delayed adapted management and influenced outcome. Facing the pandemic, several countries, notably in Europe and North America, implemented sanitary measures to limit epidemic spreading, including quarantine for patients with Covid-19 and contacts, and temporary lockdown. Although important at a collective level, these measures may hamper adequate individual management. Fear of nosocomial SARS-CoV-2 infection may limit medical facilities attendance. Delayed diagnoses and increased disease severity have already been reported in an adult with LS (29) and in children with various conditions (30). This underlines a specificity of pediatric neurology, in which clinical examination of children with acute symptoms remains of utmost importance, and reminds us about the limits of telemedicine in acute settings.

This case of an adolescent with dramatic LS and concurrent SARS-CoV-2 infection suggests synergistic effects of viral and bacterial infections on inflammation and coagulation activation. It also highlights the limits of collective sanitary measures and their potential influence on individual health. Ongoing studies will help increasing knowledge about SARS-CoV-2 infection pathophysiology and improving patients' management and outcome.

## Data Availability Statement

The original contributions presented in the study are included in the article/supplementary material, further inquiries can be directed to the corresponding author/s.

## Ethics Statement

Ethical review and approval was not required for the study on human participants in accordance with the local legislation and institutional requirements. Written informed consent to participate in this study was provided by the participants' legal guardian/next of kin.

## Author Contributions

MK, FM, C-JR, LD, and CM designed and conceptualized the report, acquired and interpreted patient data, drafted the manuscript for intellectual content, reviewed, and revised the manuscript. IE, PP, and RL acquired and interpreted patient data, reviewed and revised the manuscript. All authors approved the final manuscript as submitted and agreed to be accountable for all aspects of the work.

## Conflict of Interest

The authors declare that the research was conducted in the absence of any commercial or financial relationships that could be construed as a potential conflict of interest.

## Abbreviations

CSF: cerebro-spinal fluid
LS: Lemierre syndrome
MCA: middle cerebral artery
MRI magnetic resonance imaging
PCR: polymerase chain reaction.
